# CircPac: A web-based analysis toolset for exploring circular RNA data

**DOI:** 10.1016/j.dib.2025.111724

**Published:** 2025-06-02

**Authors:** Amir Hossein Foroutan, Sadra Salehi-Mazandarani, Maryam Lotfi-Shahreza, Parvaneh Nikpour

**Affiliations:** a Department of Computer Engineering, Shahreza Campus, University of Isfahan, Isfahan, Iran; b Department of Genetics and Molecular Biology, Faculty of Medicine, Isfahan University of Medical Sciences, Isfahan, Iran; c Faculty of Electrical and Computer Engineering, Tarbiat Modares University, Tehran, Iran; d Department of Psychiatry and Neurochemistry, Institute of Neuroscience and Physiology, Sahlgrenska Academy at University of Gothenburg, Gothenburg, Sweden

**Keywords:** RNA, Circular, MicroRNAs, Computational biology, Web-based analysis, Data visualization

## Abstract

Circular RNAs (circRNAs) are a type of RNAs that play crucial roles in various biological processes. Their outstanding properties such as tissue-specific expression and high resistance to exonuclease degradation make them attractive for research. However, a comprehensive analysis tool for analyzing circRNA data is still required. Here, we present CircPac, a newly developed web-based toolset that searches databases like circBase, circBank, and circRNADisease and organizes data to provide and visualize circRNAs information. Our toolset was created using the Python programming language and its libraries, such as pandas, seaborn, and the Django framework. CircPac enables users to unify the circRNA IDs and subsequently perform various bioinformatic analyses. These analyses include retrieving basic circRNA information, identifying target miRNAs, and analyzing circRNA expression changes in various diseases. Additionally, this toolset generates ready-to-publish figures of circRNA-miRNA interactions and circRNAs expression changes in diseases. CircPac is freely accessible (at https://www.circpac.ir) and offers a user-friendly platform for biologists to efficiently conduct and visualize circRNA data analyses in an appropriate format.

## Graphical abstract

**Figure:**
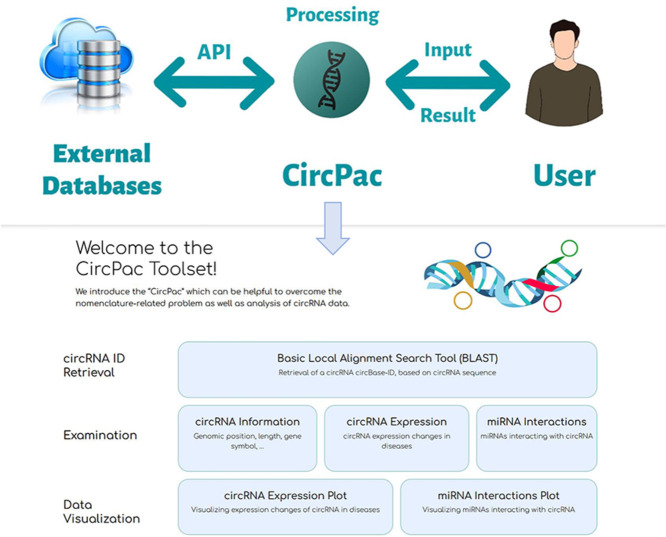

Specifications Table

**Table:** 

Subject	Bioinformatics
Specific subject area	“Development and application of a web-based toolset for analysing and visualizing circular RNA data.”
Type of data	Input data: Table, Raw, Analyzed Output data: Table, Graph, Figure, Processed
Data collection	The datasets were retrieved from the circBase, circBank, circRNADisease, and CircFunBase biological databases. The circBase, circBank, and circRNADisease data were obtained from downloadable files, while the CircFunBase data were retrieved using its API with permission. The integrated datasets encompass data from high-throughput technologies, computational analyses, and literature review data. No intervention was performed on the raw data; thus, they were taken as is from the databases with no further normalization.
Data source location	The data sources for this research are public biological databases, specifically circBase, circBank, circRNADisease, and CircFunBase. These databases house information derived from experimental assays, computational analyses, and published literature, accessible online through their respective platforms and APIs.
Data accessibility	Repository name: GitHub Data identification number: 10.5281/zenodo.14202090 Direct URL to data: https://github.com/amirhfro/circpac_data
Related research article	None.

## 1 Value of the Data

The data presented in this research are valuable to the scientific community as they provide a comprehensive toolset, CircPac, that addresses critical challenges in circular RNA (circRNA) analysis, such as the lack of standardized nomenclature. By enabling the unification of circRNA IDs from multiple databases into circBase-IDs, the data streamline the integration of information from diverse sources, making it easier for researchers to conduct bioinformatics analyses. Additionally, the tool facilitates the exploration of interactions between circRNAs and microRNAs (miRNAs), contributing to the study of competing endogenous RNA (ceRNA) networks and their roles in diseases mechanisms. Researchers can reuse the data to investigate circRNA expression changes in various diseases, which may aid in the identification of diagnostic or prognostic biomarkers. Furthermore, CircPac supports the generation of high-quality, publication-ready visualizations, including chord plots and heatmaps, making it a practical resource for presenting complex miRNA-circRNA interactions and circRNA expression changes in diseases in a clear and accessible format.

## 2 Background

**Fig. 1:**
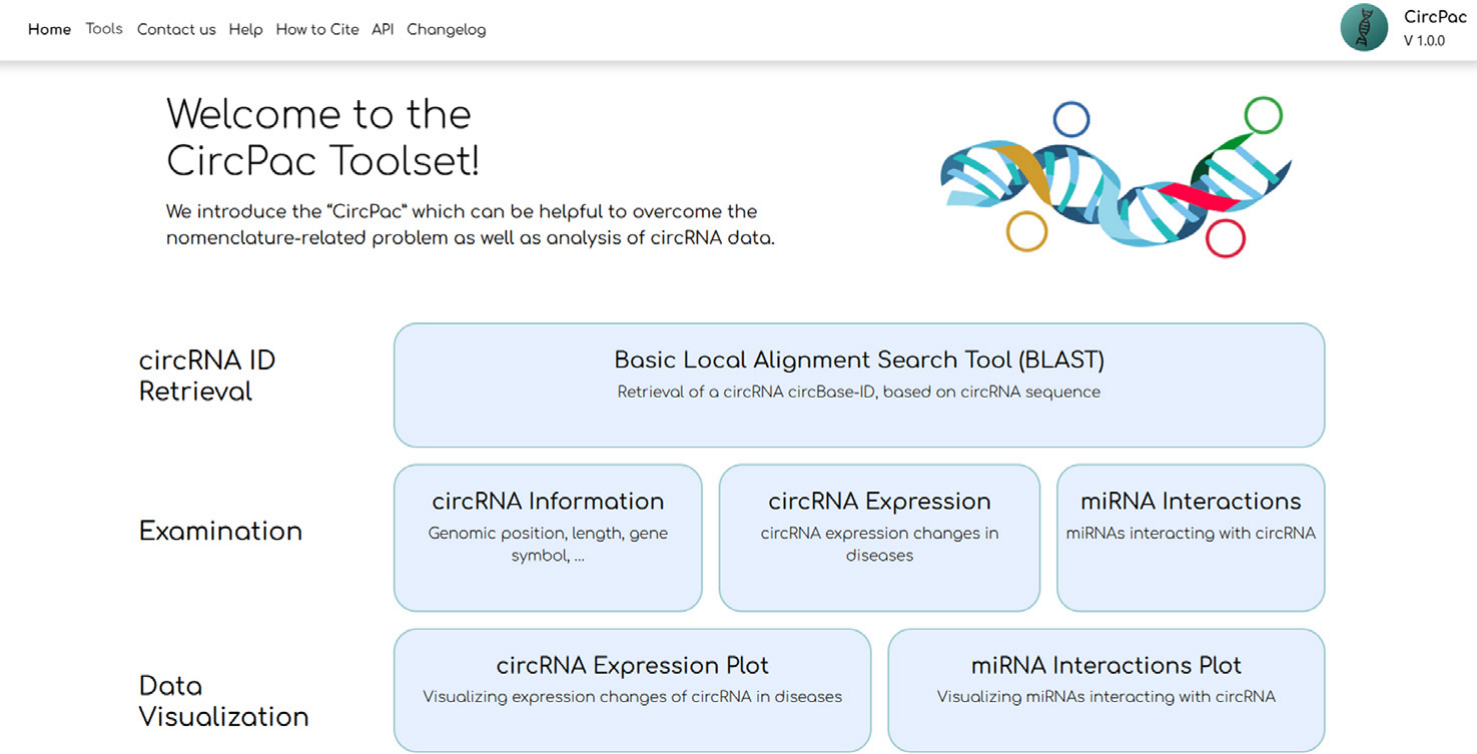
CircPac website homepage view. CircPac main features include: 1- Retrieval of a circRNA circBase-ID based on its sequence and via BLAST, 2- Examination of circRNAs including retrieval of some basic information such as genomic position, length, gene symbol, expression changes of circRNAs in diseases and miRNAs interacting with a specific circRNA and 3- Data visualization which is a graphical representation of two latter parts of the 2^nd^ feature (expression changes of circRNAs and miRNA-circRNA interactions). BLAST: Basic Local Alignment Search Tool.

Circular RNAs (circRNAs) are a class of single-stranded RNAs with a closed structure that are being produced from precursor messenger RNAs (mRNAs) based on a process called back splicing [1]. circRNAs have outstanding features such as resistance to exonucleases, high expression levels and tissue-specific expression which make them ideal targets for clinical research [2]. One of the important difficulties in dealing with circRNA data is that circRNAs are being recognized with different IDs and they do not have a unique nomenclature system yet [3]. In the current study, we introduce the “CircPac” toolset which can be helpful to overcome the nomenclature-related problem as well as analysis of circRNA-related data. This toolset provides a user-friendly environment for the users and enables them to get information in three ways: A) Retrieval of a circRNA circBase-ID, as the most used ID, based on its sequence, B) Examination of circRNAs including retrieval of some basic information for each circRNA, expression changes of circRNAs in diseases and miRNAs interacting with a specific circRNA and C) Data visualization (Fig. 1).

## 3 Data Description

CircPac provides a user-friendly environment to browse circRNAs data. Here, we present the results and discuss the characteristics and capabilities of this toolset which includes three parts: circRNAs sequences alignment for retrieval of their circBase-IDs, examination of circRNAs attributes, and circRNA data visualization.

### 3.1 circRNA sequences alignment

Several databases have been established to facilitate the analysis of circRNAs data. However, a notable challenge in circRNA data analysis arises from the absence of a standardized nomenclature system [3]. This lack of uniformity complicates tasks such as integrating circRNA data with diverse nomenclature styles or conducting analyses in databases that require specific formats for circRNA IDs. For instance, researchers may encounter difficulties when attempting to merge circRNA data from different sources or when analyzing circRNAs in databases that mandate a particular format for circRNA IDs. Recently, Chen et al. [3] presented a solution to address the challenges associated with circRNA nomenclature. They proposed a novel style of nomenclature for circRNAs that aims to provide unique identifiers for circRNAs while conveying various information about their characteristics. Although this approach appears promising for ensuring the uniqueness of circRNA names, achieving universal adoption remains a distant goal. Currently, circRNAs are assigned different identifiers based on their respective databases. Among these, the circBase-ID [4] is the most prevalent type.

Nowadays common databases of circRNAs such as circAtlas [5] and circBank [6] can take circBase-IDs. Therefore, the current toolset, CircPac, provides a way to change the various IDs of circRNAs to circBase-IDs according to their sequences. Utilizing the BLAST tool, users can unify their circRNAs nomenclature based on circBase-IDs. For this aim, whole or a part of circRNAs sequences are required. This tool serves the purpose of consolidating and standardizing the names of circular RNAs, following the format used in the circBase database. The tool operates by searching for sequences within the CircFunBase [7] database and subsequently providing the corresponding name of the RNA unit, such as “hsa_circ_0000168.” Its working method involves searching the database for matching sequences and retrieving the associated circBase-IDs.

### 3.2 Examination of circRNAs attributes

**Fig. 2:**
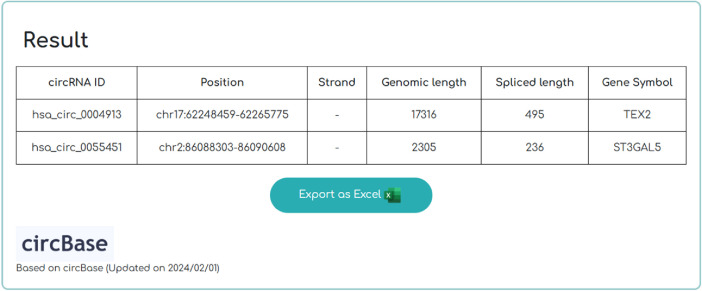
circRNA basic information output using CircPac. Basic information of two circRNAs as input of CircPac has been shown including the chromosomal position of circRNAs, the strand of DNA producing them, the length of circRNAs (genomic length) and after (spliced length) splicing, and the genes encoding them.

CircPac also provides a list of basic information about circRNAs (Fig. 2). The columns of Fig. 2 provide information in a sequential order. Starting from the left, they include details about (1) the circRNA circBase-ID; (2) its position in the genome; (3) the direction from which the circular RNA is transcribed; (4) the length of the genomic region; (5) the length of the spliced form (6), and the symbol representing the gene from that the circRNA originates.

CircPac also enables users to get circRNA expression changes in diseases which can be reached through the “circRNA Expression” tool. To this aim, only a list of circRNAs based on circBase-ID is required. Many circRNAs have been reported as differentially-expressed RNAs in diseases. For example, cir_ITCH and circHIPK3 are downregulated in gastric cancer (GC) [8]. These expression changes of circRNAs in pathological states indicate their critical roles underlying the progression of disorders. Besides, they may be introduced as diagnostic/prognostic biomarkers in the future.

Another feature of CircPac which can be reached through the “miRNA Interactions” tool is to retrieve a list of miRNAs interacting with a specific circRNA. The competition between circRNAs and other types of RNAs for miRNA binding sites gives rise to ceRNA axes and networks, which represent an emerging area of interest in non-coding RNA research [9,10]. In one of these studies, Guan et al. [11], identified differentially-expressed circRNAs, miRNAs and mRNAs between tumoral and non-tumoral GC samples. They used related online databases to identify the interaction between differentially-expressed RNAs and then established a ceRNA network in GC. Furthermore, they constructed a protein-protein interaction (PPI) network and by analysis of its hub genes they identified five critical circRNA-miRNA-mRNA regulatory axes in this type of cancer. CircPac can be utilized as an advantageous tool in such studies by retrieving the list of miRNAs interacting with circRNAs as a table as well as visualizing these interactions in the style of a chord plot.

### 3.3 circRNA data visualization

**Fig. 3:**
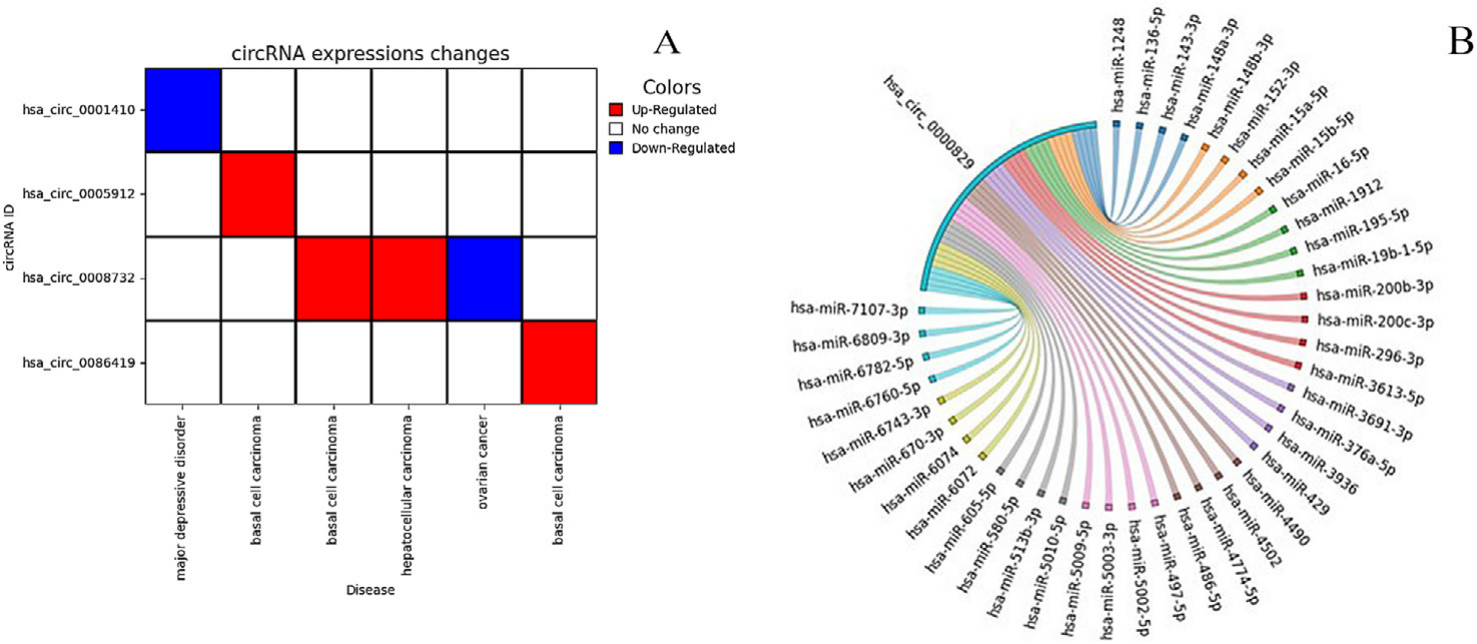
Visualization of circRNA data using CircPac. A) Heatmap plot indicating expression changes of circRNAs in diseases. X and Y axes represent diseases and circRNAs, respectively. Up-regulated circRNAs are shown with red and down-regulated circRNAs are shown with blue colors. circRNAs with no expression changes are indicated with white. B) A chord plot indicating miRNAs interacting with one circRNA (hsa_ circ_0000829).

Visualization of circRNA data analyses is of high interest to researchers. Many circRNAs are differentially-expressed in human diseases. By using the “circRNA Expression Plot” tool, users can illustrate expression changes of circRNAs in diseases as a heatmap diagram (Fig. 3A). The heatmap shows up- and down-regulated circRNAs previously reported in diseases. Furthermore, miRNA-circRNA interactions can be plotted using the “miRNA Interactions Plot” tool based on a chord plot (Fig. 3B).

## 4 Experimental Design, Materials and Methods

This section begins by detailing the data collection and implementation methods, specifically the acquisition and processing of data from biological databases like circBase [4], circBank [6], circRNADisease [12], and CircFunBase [7], as outlined in Section 4.1. Following this, Section 4.2 introduces CircPac's key features, including circRNA ID retrieval tools, examination, and visualization. The CircPac alignment tool is discussed in Section 4.2.1, highlighting its use of Basic Local Alignment Search Tool (BLAST) for sequence alignment and circBase-ID retrieval. Section 4.2.2 examines circRNA attributes, focusing on retrieving basic information, expression data, and circRNA-miRNA interactions. Finally, Section 4.2.3 addresses data visualization, where Python libraries such as PyCirclize and Seaborn are utilized to create heatmaps and chord diagrams, offering a visual interpretation of the data.

### 4.1 Data collection and implementation

**Fig. 4:**
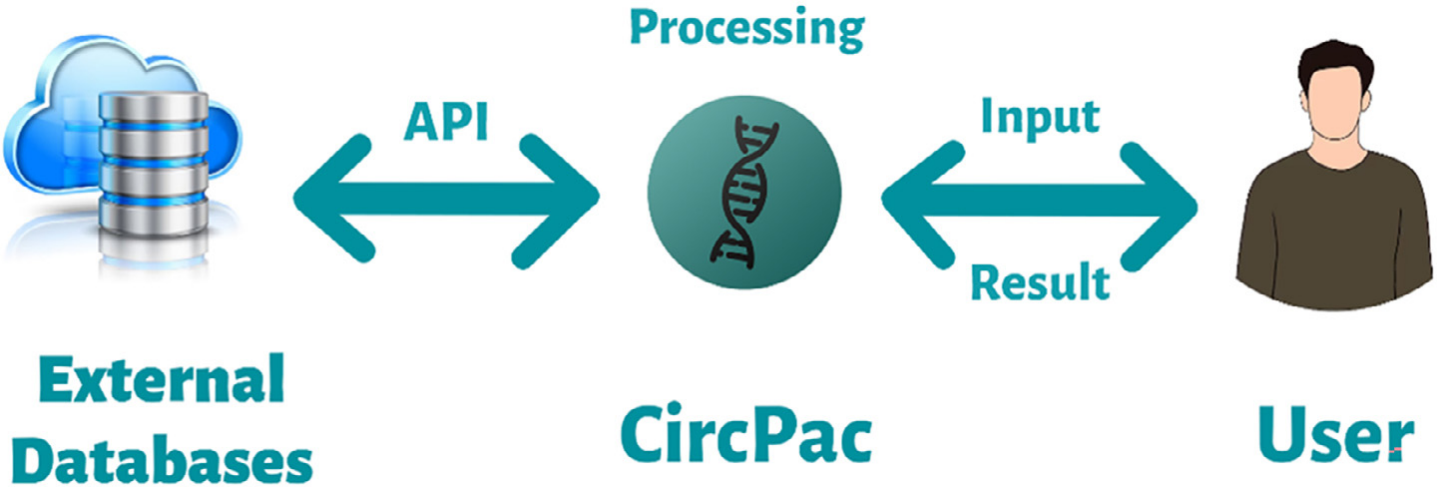
A flow diagram showing the interaction between the user, CircPac, and external databases, via an Application Programming Interface (API).

The data for this study originates from reputable biological databases, specifically circBase, circBank, circRNADisease, and CircFunBase. Of note, the word “database” here refers to the collection of biological data obtained from experiment assays including high-throughput technologies, computational analyses, and published literature. We utilized the downloadable files from circBank, circRNADisease, circBase databases, and for CircFunBase, we accessed its data through the respective Application Programming Interface (API) (The permission was taken through contacting via email). Our analysis and comparisons were conducted with the data obtained without any intervention. To acquire basic information about circRNAs, including genomic positions, strands, genomic length, spliced length, and gene symbols, we utilized circBase. We furthermore extracted details on miRNA-circRNA interactions from circBank and gathered circRNA expression data from circRNADisease (Fig. 4).

CircPac webpage (https://circpac.ir), is implemented using HTML/CSS/JS in the front-end and Python programming language [13] and Django framework [14] as the back-end. Python libraries including “Circos-Plot” [15] and “Seaborn” [16] are used to visualize the circRNA-miRNA interactions and circRNA expressions, respectively.

### 4.2 CircPac key features

CircPac presents users with a trio of analytical tools encompassing circRNA ID retrieval, circRNA examination, and data visualization. This comprehensive toolset serves as a valuable resource for retrieving a circRNA's circBase-ID based on its sequence, obtaining information about circRNAs such as genomic position, length, gene symbol, etc., examining expression changes of circRNAs in diseases, and visualizing the miRNAs that interact with a specific circRNA. The automation of these processes not only enhances efficiency but also saves time, thereby facilitating more in-depth investigations in the field of circRNAs.

#### 4.2.1 CircPac alignment tool

In this section, the BLAST functionality is present. It can handle single or multiple circRNA sequences, conducting searches in the CircFunBase database one by one and providing the corresponding circBase-ID for each query. The output, which encompasses a column containing the circBase-IDs of circRNAs, is then presented to the user. CircPac BLAST reads data from input fields utilizing the pandas Python library [17] to extract circBase-IDs. Subsequently, the circRNA sequences are dispatched to the CircFunBase BLAST web service. To interface with the CircFunBase web service, we employed their respective API. The outcomes from BLAST are processed using the BeautifulSoup [18] Python library to extract relevant information. As an output, a new Excel file is generated and made available for the user to download.

#### 4.2.2 Examination of circRNAs attributes

We designed the circRNA information tool to explore the circBase website for retrieving basic circRNA information. Operating on circRNA circBase-IDs as input, this tool executes a database query, retrieves outcomes, and extracts pertinent basic circRNA information from the database. If the user gives multiple circRNA circBase-IDs as input in this tool, each ID must be separated by commas. Similarly, we designed the circRNA expression tool which utilizes circBase-IDs to retrieve circRNA expression information from the circRNADisease database. This tool processes input circBase-IDs and utilizes an Excel file that includes experimentally supported circRNA-disease association data. This Excel file was downloaded from the circRNADisease database. Each circBase-ID from the user input list is cross-referenced with the circRNA (circBase-)IDs in this Excel file, then the matching results will be filtered and converted into JSON format for presentation as a table to the user on the front-end. This table can be downloaded as an Excel file if needed. Furthermore, we created a circRNA-miRNA interaction tool to probe the circBank database for circRNA-miRNA interactions using designated circRNA circBase-IDs. The tool gathers input (circBase-)IDs and proceeds to identify corresponding miRNA IDs from the circBank database. The outcomes, encapsulating circRNA-miRNA interactions, are presented to the user and can be downloaded as an Excel file.

#### 4.2.3 Data visualization

This set of tools serves the purpose of displaying and visualizing the analyzed data, utilizing two Python modules: "PyCirclize" and "Seaborn". PyCirclize is employed in the "miRNA Interactions Plot" tool to showcase circRNA and miRNA interactions in a circular chord diagram style. Seaborn is utilized to generate heatmap diagrams, offering insights into circRNA expression changes in various diseases. The circRNA Expression Plot tool is designed for data visualization based on circRNA circBase-ID as input. The resulting data is presented as a heatmap diagram, illustrating circRNA expression patterns across different diseases, and can be saved as a PNG image. In the miRNA Interactions Plot tool, a chord diagram is generated based on the input circRNA circBase-IDs. The tool retrieves the input (circBase-)IDs, reads a CSV file containing circRNA-miRNA interaction data, and iterates over the DataFrame rows. It retrieves corresponding miRNA IDs, storing the results in separate lists. A dictionary is then created to combine circRNA (circBase-)IDs and their associated miRNA IDs, forming a new data frame. The tool generates a binary interaction matrix through one-hot encoding, further processed to create a chord plot using the PyCirclize library. The resulting chord plot is returned as an HTTP response in the PNG format, providing a visual representation of circRNA-miRNA interactions.

## Limitations

The quality and accuracy of the data depend on the reliability of the source databases, such as circBase, circBank, circRNADisease, and CircFunBase, which may contain inconsistencies or incomplete records. Furthermore, the curated datasets used for analysis may carry inherent biases, such as an overrepresentation of certain disease-related circRNAs due to their popularity in research, which could affect generalizability. Finally, the reliance on API access for certain datasets may occasionally encounter technical constraints or updates that could impact data retrieval.

## Ethics Statement

The authors confirm that they have read and adhered to the ethical requirements for publication in Data in Brief. The current work does not involve human subjects, animal experiments, or any data collected from social media platforms. The research utilized publicly available data from biological databases, and no ethical concerns related to data collection or usage were encountered.

## CRediT Author Statement

**Amir Hossein Foroutan:** Methodology, Software, Formal analysis, Investigation, Resources, Data curation, Writing – original draft, Visualization, Validation. **Sadra Salehi-Mazandarani:** Conceptualization, Resources, Writing – original draft, Validation. **Maryam Lotfi-Shahreza:** Methodology, Project administration, Writing – review & editing, Validation. **Parvaneh Nikpour:** Supervision, Writing – review & editing, Validation.

## Declaration of Competing Interests

The authors declare that they have no known competing financial interests or personal relationships that could have appeared to influence the work reported in this paper.

## Data Availability

GitHubcircpac data (Reference data).
